# Correlation of serum delta-like ligand-4 level with the severity of diabetic retinopathy

**DOI:** 10.1186/s12902-021-00814-6

**Published:** 2021-08-06

**Authors:** Yan Zhu, Jingcheng Hu, Xuan Du, Qionglei Fang, Yingyi Zhou, Ke Chen

**Affiliations:** 1 Department of Endocrinology, The First Affiliated Hospital of Soochow University, No.188 Shizi Road, Suzhou, China; 2 Department of Thoracic Surgery, The First Affiliated Hospital of Soochow University, No.188 Shizi Road, Suzhou, China

**Keywords:** Type 2 diabetes mellitus, Diabetic retinopathy, Delta-like ligand-4, Independent risk factor

## Abstract

**Background:**

Diabetic retinopathy (DR) is one of the most serious microvascular complications of type 2 diabetes mellitus (T2DM). Delta-like ligand-4 (DLL4) maintains the normal physiological microenvironment of the retina. However, the relationship between the level of DLL4 and the severity of DR remains unclear.

**Methods:**

We retrospectively analyzed serum DLL4 levels and other laboratory and clinical data in 94 T2DM patients (35 patients without DR [NDR], 32 with non-proliferative DR [NPDR], 27 with proliferative DR [PDR]), and 30 healthy controls.

**Results:**

The serum DLL4 level was significantly greater in the NDR group (43.38 ± 16.23 pg/mL), NPDR group (56.57 ± 25.89 pg/mL), and PDR group (74.97 ± 25.28 pg/mL) than in the healthy controls (29.9 ± 8.92 pg/mL; all *p* < 0.05). Among T2DM patients, the level of DLL4 increased as the severity of DR increased (*p* < 0.05). Logistic regression analysis demonstrated that DR was positively associated with DLL4, glycosylated hemoglobin (HbA1c), fasting blood glucose (FBG), and duration of T2DM (all *p* < 0.05). Consistently, receiver operating characteristic (ROC) curve analysis also indicated that DLL4 was a potential candidate biomarker for identifying the severity of DR.

**Conclusions:**

T2DM patients, especially those with DR, have increased serum levels of DLL4. DLL4 may be used as a biomarker and an independent risk factor for DR, and targeting DLL4 may be a potential therapy in patients with DR.

**Supplementary Information:**

The online version contains supplementary material available at 10.1186/s12902-021-00814-6.

## Background

Diabetes mellitus (DM) is a metabolic disease that has multiple etiologies and is characterized by hyperglycemia and the abnormal metabolism of glucose, fat, and protein due to insufficient insulin secretion and/or insulin resistance [1, 2]. According to the 2017 International Diabetes Federation (IDF) Global Diabetes Survey, about 425 million people worldwide suffer from DM, and 114 million adults (20–79 years-old) in China have DM, accounting for about 10 % of all adults in China [3]. Thus, China has more adults with DM than any other country.

Diabetic retinopathy (DR), one of the most serious microvascular complications of DM, is the main cause of blindness in working-age individuals [4], and is present in about one-third of DM patients. In China, the risk of blindness in DM patients is about 25-times higher than in non-DM patients, and the severity and risk of DR increases as DM progresses [5] During the early stages of DR, patients are usually asymptomatic. About 25 % of patients already have early-stage DR at the initial diagnosis of DM, and 60 % of patients who had type 2 DM (T2DM) for more than 20 years have fundus lesions of different severities [6]. Without prompt treatment, DR can seriously impair vision and eventually lead to blindness. Therefore, the early prevention and diagnosis and the timely and effective treatment of DR are very important for these patients.

Delta-like ligand 4 (DLL4), which is a ligand of the notch receptor, is a type I transmembrane protein composed of 685 amino acids whose gene is located on chromosome 15Q14 [7]. Notch maintains the normal physiological microenvironment of the retina and participates in the generation of retinal neovascularization under hypoxia. DLL4 is one of several ligands of notch proteins, its expression is greater in the presence of physiological and pathological neovascularization, and it plays an important regulatory role in retinal angiogenesis [8]. DLL4-mediated notch signaling is essential for islet function and insulin secretion in humans, but the relationship between DLL4 and DR remains incompletely understood.

In the present study, we examined the association of the serum level of DLL4 with the occurrence and progression of DR, and assessed the possible use of serum DLL4 as a risk factor for DR and a potential target for treatment of DR. Thus, we compared the clinical characteristics and serum levels of DLL4 in healthy controls and in patients with T2DM who had different severities of DR.

## Methods

### Subjects

From March 2019 to June 2019, 94 patients from the First Affiliated Hospital of Soochow University (Suzhou, China) were enrolled, all of whom were diagnosed with T2DM based on the 1999 WHO diagnostic criteria. According to the 2003 International Clinical Diabetic Retinopathy and Diabetic Macular Edema Severity Scales [9, 10], the patients were divided into 3 subgroups according to the clinical results (dilatation and fundus photography or fundus fluorescein angiography). There were 35 patients with no diabetic retinopathy (NDR), 32 patients with non-proliferative retinopathy (NPDR, with mild fundus lesions), and 27 patients with proliferative diabetic retinopathy (PDR, with neovascularization). An additional 30 healthy subjects without DM were selected from the physical examination center of the same institution as normal controls (NCs).

The main inclusion criteria were: diagnosis of diabetes for a certain course; microaneurysms, exudation, blood leakage in the fundus. For non-proliferative stage, retinal dot and blot hemorrhage and hard exudation or cotton wool spots are the clinical manifestations. For proliferative stage, neovascularization occurs in the retina, which seriously affects vision and life quality. The main exclusion criteria were: severe complications of DM; type 1 DM, gestational diabetes, or any other type of DM; severe functional impairment or disorder of the liver or kidneys; acute and/or chronic infection, such as intestinal infection, urinary tract infection, and respiratory tract infection; recent trauma, surgery, or complication from a cardiovascular or cerebrovascular event or other stress; autoimmune disease; another fundus disease, such as macular degeneration; and chronic smoking or drinking of alcohol. All subjects provided informed consent. This study was approved by the ethics committee of the First Affiliated Hospital of Soochow University (2019-059), and all methods were performed in accordance with the relevant guidelines and regulations.

### General and laboratory data

Age, sex, duration of DM, body mass index (BMI), diastolic blood pressure (DBP), systolic blood pressure (SBP), and other general information were recorded. Fasting venous blood was obtained before treatment, and serum and plasma were separated by centrifugation. The serum concentration of DLL4 was detected using an ELISA according to the manufacturer’s instructions (Blue Gene). A Hitachi7600 automatic analyzer was used to measure the level of fasting blood glucose (FBG) by the glucose oxidase method; and the levels of total cholesterol (TC), triglycerides (TG), high-density lipoprotein cholesterol (HDL-C), and low-density lipoprotein cholesterol (LDL-C) by an enzymatic method. The concentration of glycated hemoglobin (HbA1c) was determined by high pressure liquid chromatography using the Bio-Rad Variant II glycosylated hemoglobin detector. Morning urine was collected in a disposable container without contamination. Urinary protein was measured using an automatic analyzer, and the protein-creatinine ratio was calculated for assessment of proteinuria.

### Statistical analysis

All data were recorded and collated using Microsoft Excel, and statistical analyses were performed using SPSS version 23.0. Count data were presented as numbers and compared using the chi-square test, and continuous data were presented as means and standard deviations. Analysis of variance was used to compare the different groups. The relationships of DLL4 with other clinical indicators were determined using Pearson correlation analysis. A logistic regression model was used to identify factors associated with DR. A receiver operating characteristic (ROC) curve was used to determine the optimal cut-off value of DLL4 for the diagnosis if DR. A P-value below 0.05 was considered significant.

## Results

### General characteristics of the four groups

The four groups had no significant differences in gender or age (all *p* > 0.05; Table 1). The PDR group had a longer duration of T2DM than the NPDR group, and both groups had longer durations of T2DM than the NDR group (all *p* < 0.05). All T2DM groups together had a significantly greater BMI than the NC group (*p* < 0.05), but the 3 T2DM groups had no significant differences in BMI. Analysis of SBP indicated that all T2DM groups together had a greater level than the NC group (*p* < 0.05); the differences between the NDR and NPDR groups and between the NPDR and PDR groups were not statistically significant; but the NDR group had a significantly lower level than the PDR group (*p* < 0.05). All T2DM groups together had a greater DBP than the NC group (*p* < 0.05), but the 3 T2DM groups had no significant differences in DBP.

**Table 1:** Baseline clinical characteristics of participants in the four groups

Index	NC	NDR	NPDR	PDR
Number	30	35	32	27
Age (years)	53.0 ± 10.6	55.9 ± 12.8	53.9 ± 9.4	56.0 ± 13.6
Sex (Male/female)	16/14	19/16	18/14	12/15
Duration of diabetes (years)	–	6.54 ± 5.01	10.87 ± 6.17^b^	16.33 ± 8.02^bc^
BMI (kg/m^2^)	22.70 ± 1.85	24.31 ± 3.72^a^	25.09 ± 3.10^a^	25.07 ± 2.88^a^
SBP (mmHg)	122.27 ± 18.41	131.66 ± 15.35^a^	138.12 ± 20.03^a^	144.73 ± 18.50^ab^
DBP (mmHg)	73.6 ± 12.83	82.48 ± 11.47^a^	88.59 ± 13.48^a^	88.31 ± 1.50^a^

^a^: Significantly different from NC; ^b^: Significantly different from NDR; ^c^: Significantly different from NPDR

### Biochemical indexes of the four groups

All T2DM groups together had greater levels of HbA1c, TC, TG, LDL-C, and FBG and a lower level of HDL-C than the NC group (all *p* < 0.05; Table 2). The NPDR and PDR groups had greater levels of HbA1c, TG, and urinary protein/creatinine ratio than the NDR group, and the PDR group had greater levels of HbA1c, TG, and urinary protein/creatinine ratio than the NPDR group (all *p* < 0.05). The PDR group had a greater FBG than the NDR and NPDR groups (all *p* < 0.05). However, the NDR and NPDR groups had no significant difference in FBG, and the three T2DM groups had no significant differences in TC, HDL-C, and LDL-C.

**Table 2:** Laboratory data of participants in the four groups

Index	NC	NDR	NPDR	PDR
HbA1C (%)	5.31 ± 0.22	7.96 ± 1.37^a^	9.34 ± 1.78^ab^	10.48 ± 1.99^abc^
TC (mmol/L)	3.99 ± 0.47	4.57 ± 1.02^a^	4.74 ± 1.31^a^	4.94 ± 1.50^a^
TG (mmol/L)	1.09 ± 0.30	1.51 ± 0.98^a^	2.15 ± 1.10^ab^	3.50 ± 2.76^abc^
HDL-C (mmol/L)	1.34 ± 0.31	1.12 ± 0.28^a^	1.01 ± 0.27^a^	0.98 ± 0.45^a^
LDL-C (mmol/L)	2.23 ± 0.38	2.76 ± 0.89^a^	2.93 ± 1.09^a^	3.02 ± 0.99^a^
FBG (mmol/L)	5.20 ± 0.34	8.22 ± 3.3^a^	8.85 ± 2.27^a^	10.02 ± 1.75^abc^
Urine protein/creatinine	–	0.11 ± 0.11	0.34 ± 0.39^b^	1.51 ± 0.87^bc^
DLL4 (pg/mL)	29.9 ± 8.92	43.38 ± 16.23^a^	56.57 ± 25.89^ab^	74.97 ± 25.28^abc^

^a^: Significantly different from NC; ^b^: Significantly different from NDR; ^c^: Significantly different from NPDR

The NDR, NPDR, and PDR groups each had a higher DLL4 level than the NC group (all *p* < 0.01). In addition, the NPDR group had a higher DLL4 level than the NDR group (*p* < 0.05), and the PDR group had a higher DLL4 level than the NDR group and the NPDR group (both *p* < 0.01).

### Correlation of DLL4 with other clinical indicators

**Fig. 1:**
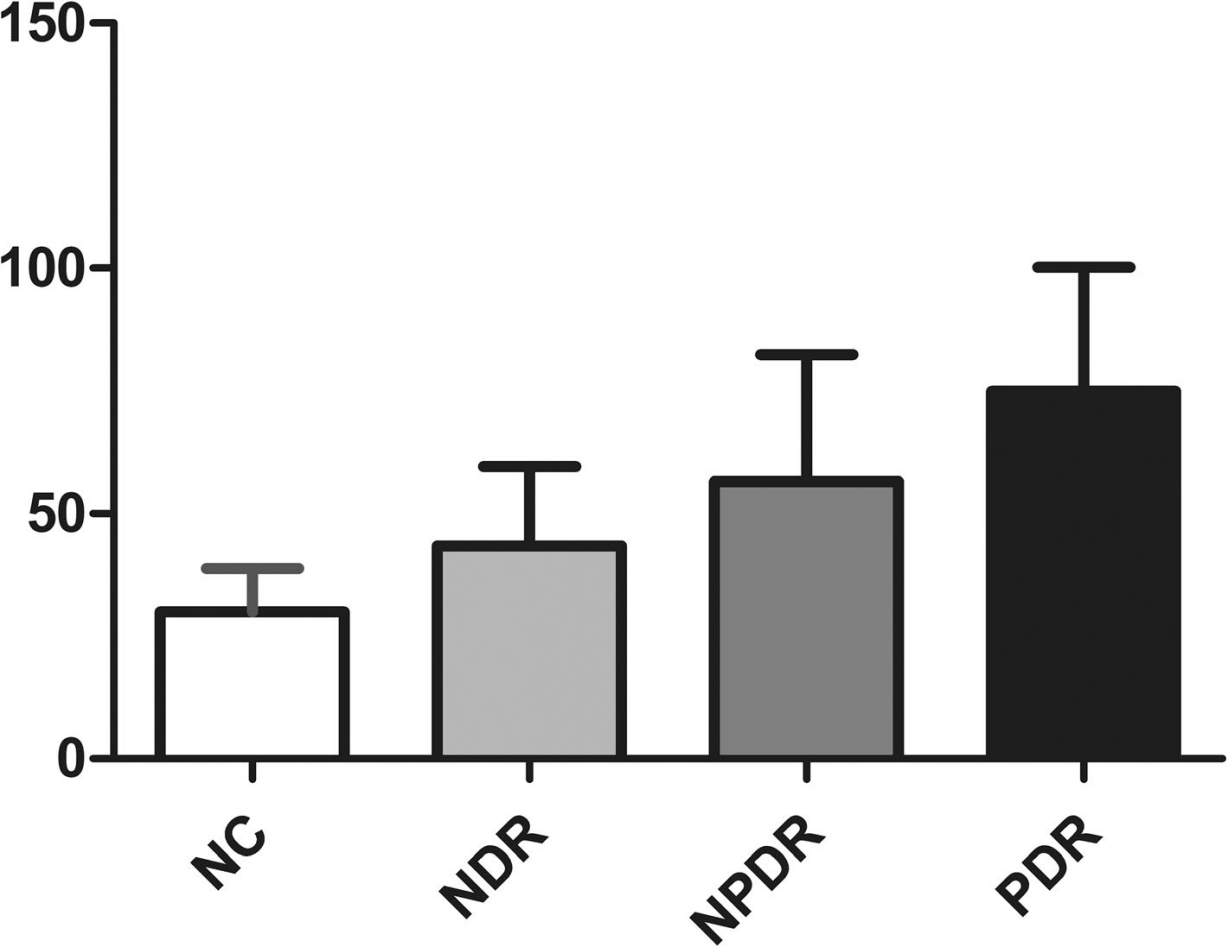
Serum concentration of DLL4 in each group. a: significantly different from the NC group; b: significantly different from the NDR group; c: significantly different from the NPDR group

Pearson correlation analysis of all NC and T2DM participants indicated that the DLL4 level was positively associated correlated with BMI, SBP, HbA1c, FBG, TC, TG, and the ratio of urinary protein and creatinine, and negatively associated with HDL-C (all *p* < 0.05; Table 3; Fig. 1).

**Table 3:** Correlation of DLL4 with other clinical indicators in T2DM participants

Index	*r*	*p*
Sex	−0.019	0.835
Age	−0.036	0.695
HDL-C	−0.193	**0.032**
Duration of diabetes	0.191	0.066
DBP	0.161	0.075
LDL-C	0.098	0.279
BMI	0.177	**0.049**
SBP	0.218	**0.016**
HbA1c	0.529	**0**
FBG	0.492	**0**
TC	0.209	**0.02**
TG	0.323	**0**
Urine protein/creatinine	0.177	**0.015**

### Use of DLL4 for identification of DR

We then examined the effect of the of duration of diabetes, SBP, HbA1c, and DLL4 (independent variables) on DR (dependent variable). Multivariate logistic regression analysis indicated that each of these independent variables was an were independent risk factor for T2DM with DR (all *p* < 0.05; Table 4), and DDL4 was also an independent risk factor for T2DM with PDR (Supplementary Table 1). Notably, the level of DLL4 was greater in patients than in healthy controls.

**Table 4:** Multivariate logistic regression analysis of factors associated with diabetic retinopathy

	unadjusted			adjusted		
**Variate**	***p***	**OR**	**95 %CI**	***p***	**OR**	**95 %CI**
DLL4	**0.000**	1.046	1.020,1.073	**0.008**	1.108	1.027, 1.195
Duration of T2DM	**0.000**	1.179	1.083,1.282	**0.007**	1.804	1.178, 2.764
FBG	**0.000**	1.502	1.214,1.859	**0.013**	1.583	1.108, 2.263
HbA1c	**0.000**	1.973	1.412,2.756	**0.025**	1.793	1.091, 3.246
BMI	0.536	0.722	0.257,2.026	0.118	0.788	0.584, 1.062
SBP	0.525	1.022	0.956,1.092	0.224	1.029	0.983, 1.076
TC	0.055	0.028	0.001,1.082	0.068	0.5	0.002, 1.243
TG	0.230	0.730	0.331,1.278	0.072	3.838	0.889, 3.017
LDL-C	0.125	5.572	0.437,1.562	0.088	4.272	0.676, 1.236
HDL-C	0.249	1.710	0.053,9.633	0.96	0.909	0.022, 7.121
Urine protein/creatinine	0.073	1.710	0.546,4.606	0.057	1.116	0.810, 3.627

**Fig. 2:**
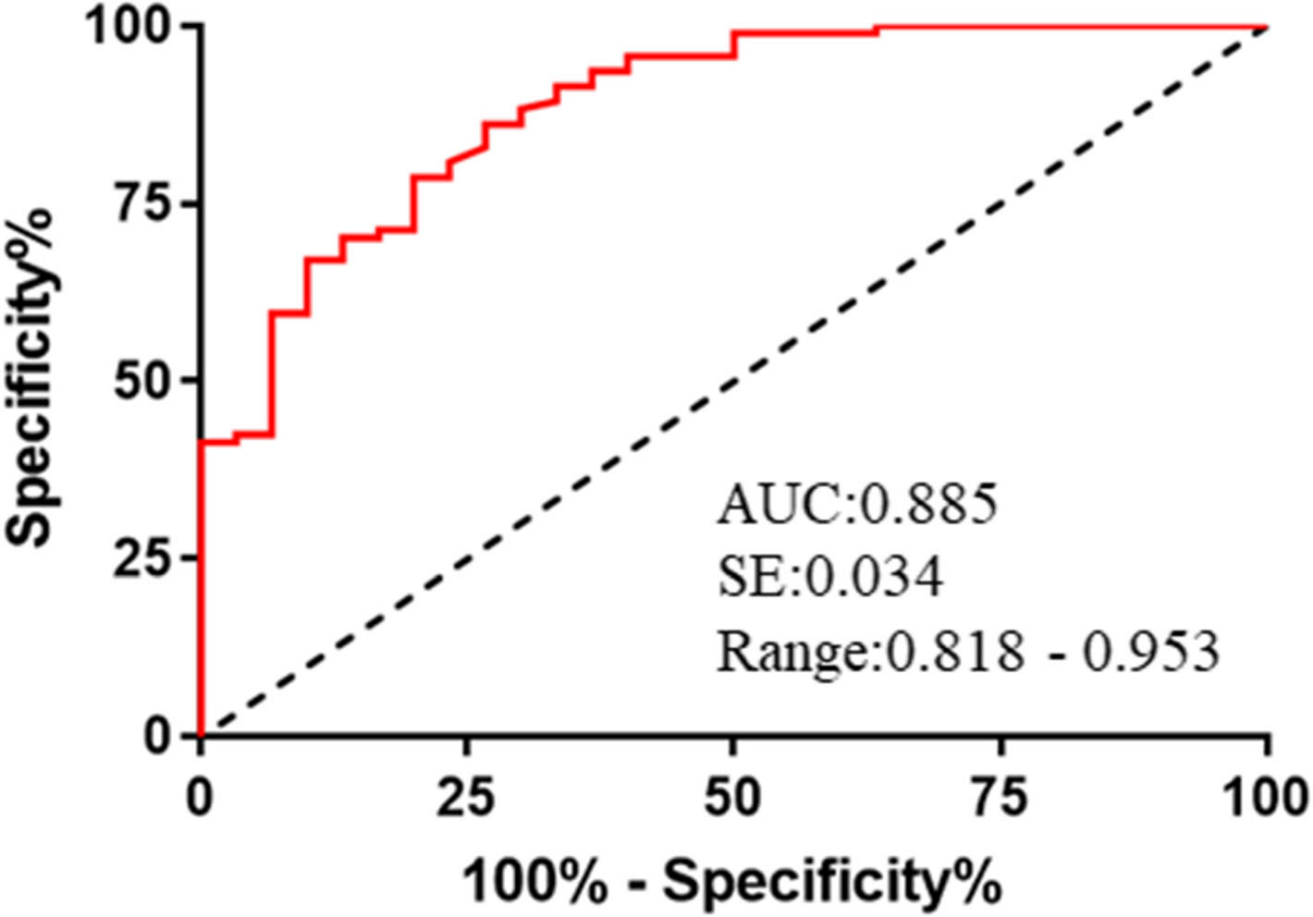
Use of serum DLL4 level for diagnosis of DR (ROC curve analysis). Patients in the NDR group were compared with those in the NPDR and PDR groups

To evaluate the possible use of DLL4 for the diagnosis of DR in clinical settings, we also performed receiver operating characteristic (ROC) analysis (Fig. 2). The results indicated excellent diagnostic performance, with an area under the curve (AUC) of 0.885 (SE: 0.034; range: 0.818–0.953).

## Discussion

The pathogenesis of DR is complex and is related to alterations in multiple pathways and molecules. These alterations include the formation of glycosylated end products, oxidative stress, up-regulation of matrix metalloproteinase, production of multiple growth factors, and secretion of adhesion molecules, although the specific mechanism is still not fully understood [1, 11]. The formation of new blood vessels during the pathological process of PDR is generally believed to be related to the production of retinal angiogenic factors caused by localized ischemia and hypoxia [12]. The most important marker of PDR is fundus neovascularization, defined by the formation of a large number of fibrous vascular membranes, and this can lead to vitreous hemorrhage and the occurrence of late traction retinal detachment [13]. Thus, fundus lesion with neovascularization caused by hypoxia is the most clinically significant alteration in DR.

Patients with PDR have impaired retinal microvascular cells, and this leads to retinal microvascular occlusion and the formation of non-perfusion areas, followed by ischemia and hypoxia in local tissues. There is evidence that hypoxia is an important initiator of the notch signaling pathway in DR [14]. Recent studies identified biomarkers and risk associated with retinal disorders in patients with DM, including C1q/TNF-related protein (CTRP3), lipasin, and microRNA-211, and these molecules may be novel therapeutic targets for the prevention or treatment of DR [15–18]. Our study showed that an elevated serum DLL4 level was an independent risk factor for DR. Other studies of the development of neovascularization in PDR patients reported that the DLL4/notch1 signaling pathway acted on the endothelium to regulate vascular remodeling, reduced the number of vascular buds and collaterals, and promoted the differentiation and maturation of neovascularization [14, 19, 20]. DR is a progressive complication of DM, and long-term hyperglycemia is the main cause of the onset and progression of DR. More specifically, a long-term high level of blood glucose and abnormal hemodynamics lead to retinal blood vessel deformation, endothelial cell damage, and damage of the blood-retinal barrier, and these all increase retinal vascular permeability and retinal leakage as the disease progresses. Notably, these pathological changes are sustained by a high level of blood glucose [21, 22]. As a result, severe vitreous hemorrhage and retinal detachment may occur. These previous findings are consistent with our current findings that FBG, HbA1c, and duration of T2DM were independent risk factors for DR.

DR fundus lesions are characterized by increased vascular permeability, formation of soft tissue ischemia, leakage from new blood vessels, bleeding, and other alterations. A prolonged high level of blood glucose can cause microvascular abnormalities, and patients with DM are often characterized by dyslipidaemia and clumping of blood cells, and thus leading to microcirculation disturbances and propel DR forward [14]. All of these pathological changes can contribute to an increased level of DLL4. Our study suggests that DLL4 plays a role in the onset and progression of DR, and can serve as a potential biomarker for assessing the risk and severity of DR. However, there are some limitations in the present study. Firstly, the level of DLL4 is also increased in many other diseases, such as infections by various pathogens, cardiovascular disease, kidney disease, tumors, and vasculitis [23–25]. Secondly, animal studies indicated that inhibition of DLL4 contributed to angiogenesis, which is quite contrary to our findings in patients with T2DM [8, 26, 27]. Thus, the usefulness of DLL4 as a specific biomarker of DR in clinical settings needs to be confirmed by further studies with large sample sizes. Thirdly, we did not check the relationship between the level of DLL4 and diabetic macular edema.

## Supplementary Information

**Additional file 1:**

## Data Availability

The datasets used and/or analyzed during the current study are available from the corresponding author on reasonable request.

## Abbreviations

DR: Diabetic retinopathy.
NDR: No diabetic retinopathy.
NDPR: Non-proliferative diabetic retinopathy.
PDR: Proliferative diabetic retinopathy.
T2DM: Type 2 diabetes mellitus.
DLL4: Delta-like ligand-4.
HbA1c: Glycosylated hemoglobin.
FBG: Fasting blood glucose.
ROC: Receiver operating characteristic.
IDF: International Diabetes Federation.
BMI: Body mass index.
DBP: Diastolic blood pressure.
SBP: Systolic blood pressure.
TC: Total cholesterol.
TG: Triglyceride.
HDL-C: High-density lipoprotein cholesterol.
LDL-C: Low-density lipoprotein cholesterol.
