# Supplementary material for: Correlation of serum delta-like ligand-4 level with the severity of diabetic retinopathy
Source: BMC Endocr Disord. 2021 Aug 6;21:157. doi: 10.1186/s12902-021-00814-6 (PMC8344193; doi:10.1186/s12902-021-00814-6)
Supplement: Supplementary file 1 — Additional file 1: [file 12902_2021_814_MOESM1_ESM.docx]

**Supplementary Table 1. Multivariate logistic regression analysis of factors associated with PDR**

| Variate | *p* | adjusted OR | 95%CI |
| --- | --- | --- | --- |
| DLL4 | **0.047** | 1.075 | 1.001, 1.155 |
| Duration of T2DM | **0.034** | 1.511 | 1.031, 2.214 |
| FBG | **0.029** | 1.353 | 0.765, 2.393 |
| HbA1c | **0.021** | 3.794 | 1.218, 3.814 |
| BMI | 0.536 | 0.722 | 0.257, 2.206 |
| SBP | 0.525 | 1.022 | 0.956, 1.092 |
| TC | 0.055 | 0.028 | 0.002, 1.243 |
| TG | 0.230 | 5.730 | 0.331, 3.287 |
| LDL-C | 0.125 | 5.972 | 0.437, 2.562 |
| HDL-C | 0.249 | 4.710 | 0.054, 9.633 |
| Urine protein/creatinine | 0.051 | 1.233 | 1.031, 2.044 |
